# Relationship between Food Insecurity and Nutritional Risk among Older Adults in Poland—A Preliminary Study

**DOI:** 10.3390/nu15143232

**Published:** 2023-07-21

**Authors:** Robert Gajda, Marzena Jeżewska-Zychowicz

**Affiliations:** 1 Department of Human Nutrition, Faculty of Biotechnology and Food Sciences, Wrocław University of Environmental and Life Sciences, Chełmońskiego 37, 51-630 Wroclaw, Poland; 2 Department of Food Market and Consumer Research, Institute of Human Nutrition Sciences, Warsaw University of Life Sciences (SGGW-WULS), Nowoursynowska 159C, 02-776 Warsaw, Poland; marzena_jezewska_zychowicz@sggw.edu.pl

**Keywords:** food insecurity, nutritional risk, older adults, public health

## Abstract

Aging populations may be associated with increased nutritional risk, malnutrition, and food insecurity. This study aims to examine the relationship between food insecurity and nutritional risk, taking into account selected characteristics of the study group, and factors describing nutritional risk. It was conducted between May and July 2021, among 417 people aged 60 and older, in two regions of Poland. Questions from the SCREEN-14 questionnaire were used to assess nutritional risk. Selected questions from the HFSS questionnaire (U.S. Household Food Security Survey Module) concerning the elderly were used to assess food insecurity. A K-means cluster analysis was used to separate homogeneous clusters into food security indicators and nutritional risk factors. The Mann–Whitney U test and Kruskal–Wallis test were used to compare mean values between groups, and the Chi-square test was used to verify the differences. Two clusters were distinguished: I—“low food security and high nutritional risk” and II—“high food security and low nutritional risk”. Cluster I included people aged 60–65, and over 75, living in urban areas, living alone or with family, with unfavorable economic situations and family relationships. Cluster II was composed of people aged 71–75, who were rural residents, living with a partner, with favorable economic situations and family relations. The vast majority of nutritional risk factors were found in Cluster I and among those at high nutritional risk. The largest number of people were affected by such nutritional risk factors such as difficulty in chewing or biting, loss in appetite, skipping meals, and perceiving one’s weight as abnormal. Moreover, the group of people most significantly affected by high nutritional risk were in unfavorable economic situations, had poor family relationships, lived alone or with family, rated their health as worse than their peers, were overweight and obese, had metabolic disease, or impeding mobility. The results obtained can be applied to the planning of social and health policies for the elderly in Poland.

## 1. Introduction

Throughout the world, countries are experiencing a trend where the proportion of older people has increased and people’s lifespans have been extended [1]. In accordance with information from the results of the National Census of Population and Housing in Poland in 2021, the share of the population in the post-productive age group (60/65 years and older) accounted for 22.8 percent of the country’s inhabitants [2]. Given the growing number and percentage of elderly people and the increasingly noticeable limitations of the traditional functions of the family (economic, social, and caregiving) [1], as well as crises such as the Great Recession, COVID-19, and armed conflicts [3], it is necessary to set new social and health policy goals aimed regarding the elderly [1].

In order to define the tasks of public health policy for the elderly, it is important to build a conceptual model of the interrelationships and interactions between the health of the elderly and its determining factors. An example of such a model is the Complex Adaptive Systems (CAS) model, developed by Majowicz et al. [4], which concerns health in relation to food and diet. The CAS model suggests that a given population health outcome emerges from an underlying system of interrelated factors, including political, environmental, social, biological, and others [5]. One way to establish data for such a model is to search for the interrelationships between food consumption and the factors affecting it [4]. They include socioeconomic status [6,7], preferences [8,9], culture [10,11], policy [12,13], legislation [14,15], economics [16,17], trading [18,19], industry [20,21], environment [20,21], environment transformed by human activity [22,23], and food security [24,25]. To develop this model further, it is necessary to study the nature of the relationship between food consumption and food safety, taking into account nutritional risk, which can extend the model, and at the same time, help to understand the relationship between food consumption and food safety in the elderly population.

The relationship between food security and diet quality [26,27], and between food security, nutritional status, the incidence of malnutrition [27,28,29], and chronic diseases (diet-related) [30,31,32] have been already confirmed. In addition, global research has identified factors affecting food security for the elderly. These factors include physical functioning and physical activity [33], the distance of stores or hypermarkets from the place of residence [34,35,36], socio-economic status [37,38], social relations, social capital, and social support [39,40,41]. Older people are more predisposed to the impact of nutrition and other factors such as food security on health compared with younger demographics [42,43,44]. Among the Polish elderly, there are such dietary problems such as a low supply of vegetables and fruits, dairy products, fish, vegetable fats, and beverages, coupled with an excessive consumption of meat and meat products, sweets, fried foods, excessive sweetening, and excessive salt intake [45,46], which can markedly increase nutritional risk.

In addition to the changes associated with aging that can affect health [47], several environmental factors, including those relating to food insecurity, affect the quality of life, nutritional status, and health of older people [28,42]. Elderly people living in a community, in conjunction with various environmental factors, may be at high nutritional risk [48,49,50], leading to a deterioration in health [47,51]. Nutritional risk factors identified within this age group may include physiological changes, as follows: reduced appetite and sensory impairment; diseases and medications that interfere with nutrient intake, absorption, and metabolism; and reduced mobility, which makes it difficult to purchase food and prepare meals. Adverse social and economic circumstances affecting this age group and their nutritional risk status are as follows: financial constraints, eating alone, inability to prepare meals, and a lack of assistance with food purchases and meal preparation. Psychological factors also affect nutritional risk status, as follows: depression, sadness, and loneliness. Aspects of the physical environment, such as the location of grocery stores, availability and affordability of public transportation, and geographic isolation also affect nutritional risk status [52]. Although the available research focuses on the relationship between food insecurity and the deterioration of health in the elderly, knowledge of the interrelationship between food security and nutritional risk still needs to be developed.

As mentioned earlier, previous research assessed the relationship between food security, nutrition, and diet quality [26,27], as well as food security, nutritional status, and the incidence of malnutrition [27,28,29], in addition to chronic diseases (diet-related) [30,31,32]. However, no studies explored the relationship between food security and nutritional risk which can lead to the loss or deterioration of health if it is not addressed in time [53]. Therefore, the aim of the study was to assess the following: (1) the relationship between food insecurity and nutritional risk, taking into account group specificities and factors describing nutritional risk; and (2) the prevalence of different nutritional risk factors and their importance in determining overall risk among the elderly. The hypotheses were as follows: (1) food insecurity is associated with nutritional risk; (2) the association between food insecurity and nutritional risk is differentiated by group specificities and factors describing risk; and (3) the occurrence of nutritional risk depends on the type of factor describing it, as well as the characteristics of the group.

## 2. Materials and Methods

### 2.1. Study Design and Sample Collection

The questionnaire survey was carried out between May and July 2021, in the Świętokrzyskie and Dolnośląskie provinces in Poland. The provinces are similar in terms of their shares of people aged 60 and over (30.7% and 29.9%, respectively), which is slightly higher than the number of older people in the population (25.6%) [52]. The study sample was selected arbitrarily by inviting 21 senior organizations to participate in the study. The criteria for inclusion in the study were aged 60 and older, and they resided in the local community. Nine-hundred questionnaires were distributed to beneficiaries of senior organizations, of which, 466 were returned to the researcher upon completion. During the verification of questionnaires, 49 were rejected as they were completed inadequately. Thus, the study sample consisted of 417 people.

The study was conducted in accordance with Declaration of Helsinki [54]. Informed consent was obtained from all participants.

### 2.2. Questionnaire

Questions from the SCREEN-14 questionnaire (Seniors in the Community: Risk Evaluation for Eating and Nutrition) were used to assess nutritional risk [53]. The questionnaire included 17 questions on the occurrence of selected situations during the preceding 6 months. The questions concerned the following: (1) body weight (perception of weight, changes in weight); (2) circumstances related to food preparation and problems with food purchases; (3) eating habits (skipping meals, limiting food intake, frequency of consumption of fruit and vegetables, meat and meat substitutes, milk and dairy products, number of beverages consumed); (4) difficulties with biting, chewing, and swallowing; (5) perception of one’s appetite. In accordance with the nutritional risk assessment procedure [53], a score was assigned to each response (ranging from 0 to 4 points). A score of 2 points or less for any situation indicated the presence of nutritional risk. An overall score informing nutritional risk was calculated by adding up the points obtained, with a minimum of 0 points and a maximum of 64 points. Based on the cut-off point (50 points), two levels of nutritional risk were distinguished: low nutritional risk (50 or more points) and high nutritional risk (less than 50 points) [53]. In addition, the number of dietary risk factors (factors with a score of 2 points or less) was calculated for each respondent, then, they were categorized into 3 groups, based on the tercile distribution: 1st tercile—5 points or less; 2nd tercile—6–8 points; 3rd tercile—9 points or more.

The questionnaire included questions assessing food insecurity in elderly households, which were adapted from the HFSS questionnaire (U.S. Household Food Security Survey Module) [55]. There were four questions regarding food security concerns in the 6 months preceding the study: (1) In the last 6 months, did you fear that you would run out of food in your household for any reason?; (2) In the last 6 months, did basic foods (e.g., bread, butter, milk, eggs) run out in your household for any reason?; (3) In the last 6 months, did your average meal decrease in size for any reason?; (4) In the last 6 months, did you have to skip a meal for any reason? The nominal scales (“yes” or “no”) were applied to answer these questions. If the answer was “yes”, the study participant answered a question about the frequency of the situation using an ordinal scale (i.e., a 5-point scale, where 1 meant “daily”, 2 meant “several times a week”, 3 meant “once a week”, 4 meant “several times a month”, and 5 meant “once a month”). During analysis, the responses were aggregated into “never” (1), “rarely” (2) (several times a month or once a month), and “at least once a week” (3) (once a week, several times a week, or daily).

To characterize the study group, questions concerning the following were asked: (1) socio-demographic characteristics (gender, age, education, place of residence); (2) economic situation (below average, average, above average); (3) self-assessed health status (worse than peers, same, better than peers), which included questions on the presence of metabolic disease (no/yes), presence of a disease that impedes mobility (no/yes), and body height and weight (participants’ BMI categories were assigned in accordance with the World Health Organization [56] (i.e., normal weight (18.5 ≤ BMI ≤ 24.99 kg/m^2^), overweight (25.0 ≤ BMI ≤ 29.99 kg/m^2^), and obese (BMI ≥ 30.0 kg/m^2^)); and (4) family characteristics, which included questions on family status and an assessment of family relations (very good, good, average, bad, very bad) (Table 1).

### 2.3. Statistical Analysis

The sociodemographic characteristics of the study sample were presented using descriptive statistics. The Chi-square test was used to assess diversity between groups. The normality was verified using the Shapiro–Wilk test. A K-means cluster analysis was applied using the number of nutritional risk factors and four indicators of food security to separate homogeneous groups (clusters) [56]. The U Mann–Whitney test and the Kruskal–Wallis test were used to compare mean values between groups (two or three, respectively). A *p*-value lower than 0.05 was considered significant.

The analyses were performed using IBM Statistics SPSS, version 27.0 (IBM Corp, Armonk, NY, USA).

## 3. Results

### 3.1. Characteristics of the Study Sample

Table 1 displays the characteristics of the study sample. Of the participants, 74.8% were women and 25.2% were men. The average age was 70.8 years (SD = 6.73). More respondents (70.7%) lived in urban areas than rural areas (29.3%). More than 1/3 of respondents lived alone. The majority (59.7%) rated their health status as the same as that of their peers. Only 27.1% were of normal weight, and the rest were overweight or obese. More than half of the people surveyed (52.3%) had a metabolic disease, and 21.1% had a disease that impeded mobility. Most people described their family relations as good or very good.

### 3.2. Relationship between Food Insecurity and Nutrition Risk—Cluster Analysis

Based on declared food security concerns and the number of nutritional risk factors, two clusters were identified: I—characterized by low food security and a high number of nutritional risk factors, and II—characterized by high food security and a low number of nutritional risk factors (Table 2).

People characterized by low food security and high nutritional risk at the same time (cluster I) are primarily those aged 60–65 and over 75, living in urban areas, and living alone or with family (with or without a partner). They described their economic situation as below average and their family relationships as average at best. In addition, they had a disease that impedes mobility. Among those characterized by high food security and low nutritional risk at the same time (cluster II), the majority of respondents were aged 71–75, rural residents, they only lived with a partner, they had above average economic situations, and very good family relationships (Table 3). Gender, education, subjective health assessment, BMI, and the presence of metabolic disease did not impact membership of the clusters.

Having difficulty chewing or biting, and changes in weight without intention, did not impact cluster membership. The percentage of subjects who were characterized by other nutritional risk factors was higher in cluster I than cluster II (Table 4).

### 3.3. The Assessment of the Prevalence of Nutritional Risk Factors and Their Importance in Determining Their Severity

Among the nutritional risk factors that affected the largest group of participants in the study were difficulty chewing or biting, loss of appetite, skipping meals, and perceiving one’s weight as abnormal. On the other hand, the use of meal replacements, limited consumption of fruits and vegetables, difficulty swallowing, limited consumption of dairy products, and changes in weight without intention impacted relatively fewer people (Table 5).

Only a loss of appetite, limited consumption of dairy products, and changes in weight without intention did not discriminate between those with low and high nutritional risk. Difficulty chewing or biting, and a limited consumption of fruits and vegetables, characterized more people with a low nutritional risk than those with high nutritional risk. Other nutritional risk factors characterized a more significant proportion of people with high nutritional risk than low nutritional risk (Table 5).

There were no differences with respect to the impact of the limited consumption of fruits and vegetables or changes in weight without intention after accounting for the number of nutritional risk factors. Regarding the other factors, it was observed that as the number of nutritional risk factors increased, the percentage of people found to have them increased (Table 5).

### 3.4. Differences in the Severity of Nutritional Risk with Selected Characteristics of the Study Group

Differences in the prevalence of high and low nutritional risk were found after taking BMI, economic situation, family relationships, the presence of metabolic disease, and the presence of disease that impedes mobility into account (Table 6). A higher percentage of overweight and obese people were characterized by high nutritional risk whereas the normal weight group had more than two times as many people with low nutritional risk than high nutritional risk. The group of people with above average economic situations included fewer respondents with low nutritional risk, and groups with average and below-average economic situations were characterized by a higher percentage of people with high nutritional risk. More people with metabolic disease and impaired mobility were characterized by high nutritional risk. Very good family relationships characterized more people with low dietary risk than high dietary risk, and vice versa. More than three times as many people with high nutritional risk than low nutritional risk reported average family relationships. Higher levels of nutritional risk were found among those living alone or with family rather than just with a partner, as well as among those rating their health as worse than their peers (Table 6).

## 4. Discussion

This study assessed the relationship between food insecurity and nutritional risk. Food insecurity was estimated by taking concerns about food availability, a lack of staple foods, reduced meal sizes, and skipping meals into account [54]. Nutritional risk, on the other hand, was estimated by taking the following into account: inadequate food intake, the possibility of eating in the company of others, limitations related to difficulties in biting, chewing, and swallowing, diet, financial resources available for the purchase of food, and adapting to changes in order to function in the environment (ability to purchase food and prepare meals independently) [48,51]. The results of the study confirmed the relationship between food insecurity and nutritional risk, with low food security being associated with high nutritional risk (Cluster I), and conversely, high food security being associated with low nutritional risk (Cluster II). Clustering differed by age, place of residence, family status, and economic situation. In addition, the differences consisted of a higher prevalence of most nutritional risk factors in Cluster I, except for risk factors such as difficulty chewing and biting and changes in weight without intention. The lack of differences between the two groups concerning recent factors related to physiological functioning confirms the usefulness of using nutritional risk alongside previous approaches to explain the relationship between food intake, food security, and consequently, the health status of older adults.

Many studies to date have shown a link between food insecurity and poorer diet quality [26,27], as well as between food insecurity, nutritional disorders, and the incidence of malnutrition [27,28,29,57]. Nutritional risks refer to factors associated with the reduced quantity or improper quality of food consumed. Failure to eliminate these factors can lead to malnutrition over time [51] when combined with feelings of food insecurity. Food insecurity may be related to higher nutritional risk, as confirmed by the results of our study. Membership of clusters, in terms of food insecurity and nutritional risk, was not impacted by gender, education, self-reported health, BMI, and the presence of metabolic disease. However, the results of other studies confirm the link between food insecurity, malnutrition, and some of these characteristics, including gender [27,58,59,60], education [57,60], and body mass index [60]. At the same time, it should be noted that these results mainly come from developing countries such as Brazil, Ecuador, Peru, or Malaysia [27,57,58,60] and women, people with a lower education status, and those with a lower body mass index, experience both food insecurity and malnutrition. The lack of consistency across the results of this study compared with previous studies on malnutrition and food insecurity can be explained in two ways. Firstly, malnutrition is confirmed by specific indicators, including physiological ones, whereas food risk factors have a broader context, including self-reported indicators such as perception of body weight. Secondly, socio-demographic variation in nutritional risk factors and in feelings of food insecurity, may be highly influenced by the socio-economic situation of the country and they may be less important in developed countries [61,62].

The majority of the existing research on food insecurity in Europe is focused mainly on the situation in Western European countries, such as the United Kingdom [63], Ireland [64], Portugal [28], and Germany [65]. In Poland, considerable research has been conducted on its socioeconomic situation, including the various aspects of poverty [66,67], however, there is a shortage of studies on food insecurity, including food insecurity among the elderly [68]. Better recognized, however, are various aspects of elderly health and life conditions [69,70,71]. A high prevalence of malnutrition and the risk of its development among community-dwelling elderly people in Poland was observed [70]. As in other European studies [28,72,73], various population characteristics favored poor nutritional status, including being female, older, unmarried, living in a rural area, and living in self-reported poverty [70,71], which was also confirmed in our study. Thus, changes in socio-economic situation, including increased levels of poverty and social inequality [74], require monitoring and food policy actions to eliminate barriers to food consumption, which, in turn, will improve the nutritional and health status of older people [75]. In addition, recent research shows that during the pandemic, elderly people in Poland and Germany assessed their quality of life, well-being, and life satisfaction as being better compared with other demographic groups; this might be associated with their financial stability, resulting from their right to retirement [74]. However, self-assessment does not necessarily imply the satisfactory fulfillment of needs from a nutritional recommendation perspective. More comparative and longitudinal research is needed to understand the extent of food security problems and nutritional risk in the elderly across European countries over time. Hence, the need emerges to identify groups at risk of food insecurity and to reduce negative phenomena by taking effective actions.

Food insecurity and malnutrition often affect people of advanced ages [59], single residents [27,57], individuals living with family members or people other than a partner [76], as well as those with difficult economic situations [28,57], worse social or family relations [57,76], and those with various diseases [59,77,78]. Place of residence, food insecurity, and malnutrition were more strongly associated with rural than urban environments [57,79]; living with one’s partner protected against such consequences [57]. Such associations were also confirmed in this study, which may indicate that a nutritional risk assessment is similarly linked to feelings of food insecurity, as is malnutrition. High nutritional risk was found, among people with the following characteristics: average and poorer self-reported economic status, with less satisfactory family relationships; living alone or with family, rather than only with a partner; judging their health to be worse than their peers; and being overweight or obese, with metabolic disease and impaired mobility. These findings may confirm the link between food insecurity, nutritional risk, and consequently, malnutrition.

The results support the hypothesis that the elderly present with a social, health, and nutritional status that may make them more likely to develop malnutrition [27]. Factors contributing to malnutrition are associated with the “social helplessness” of the elderly, which hinders access to an adequate and healthy diet and the perception of its importance in the face of many other priorities [28,80,81]. In addition, there is a combination of factors affecting the health of older adults, such as the physiological repercussions of aging itself, and changes from pathological processes that affect the diet of older adults and can affect their nutritional status [57]. Moreover, older people experiencing food insecurity develop unfavorable dietary patterns that may not meet dietary recommendations [82,83]. Some researchers hypothesize that, especially in less affluent countries, being an elderly person in itself is a risk factor for food insecurity and malnutrition among those living in the community [27].

Among the nutritional risk factors that affected the largest group of participants in the study were difficulties in chewing or biting, loss of appetite, and skipping meals. Similarly, studies on malnutrition risk factors note their association with toothlessness, difficulty chewing and living, loss of appetite, and lack of independence in terms of having meals [59,77,84,85]. In our study, the prevalence of a loss of appetite did not discriminate between those characterized by low and high nutritional risk, but as the overall number of nutritional risk factors increased, the importance of appetite increased as well, which may mean that this factor may favor the presence of other risk factors. In addition, in situations where relatively few people use meal replacements, a limited appetite could translate directly to malnutrition. Acquiring alternative forms of food to mitigate the risk of malnutrition can be difficult for the elderly, mainly due to their limited financial resources, social isolation, frailty, or disability [3,78].

Meta-analyses of studies indicate that the difficulty in swallowing (dysphagia) stimulates the risk of malnutrition [77]. However, in this study, it was a factor that affected a limited number of respondents. Similarly, relatively few people limited their intake of fruits, vegetables, and dairy products; in particular, the number of people who limited their intake of dairy products was similar between the low-risk and high-risk groups, which is also positive in terms of reducing the risk of malnutrition. Previous research has found that a low dietary supply of vegetables, fruits, and dairy products characterizes the diet of the elderly [43,44]; thus, the small percentage of people limiting their intake of these products in this study cannot be considered a sufficient indicator from a nutritional risk perspective. The low consumption of fruits and vegetables among the elderly may result from, among other things, difficulties in biting and chewing. Nonetheless, the results indicate that reduced fruit and vegetable intake was more characteristic in those with low nutritional risk than high nutritional risk, whereas difficulty chewing and biting affected those with both low and high nutritional risk factors.

### Strengths and Limitations

An increasing proportion of the elderly, a rising incidence of diseases, and household food insecurity are currently fundamental public health issues [86]. The research problem addressed in this study helps understand the interrelationship between food insecurity and nutritional risk factors in the elderly; thus, the results obtained can be helpful in developing interventions aimed at improving the quality of life of this group. As opposed to diet quality, nutritional status, incidence of malnutrition, and chronic diseases, thus far, nutritional risk factors as a complex construct have only been studied to a limited extent, which can be considered a strength of the study. In addition, the participants in the study represent the “Age in place” phenomenon (i.e., those who do not permanently use institutional care). Recognizing this phenomenon is important for a number of reasons, including the living conditions, expectations, and costs of organizing institutional care for the elderly. It is also important in order to be able to anticipate changes, and above all, to counteract adverse developments in the quality of life of the elderly who remain in their own residential environment.

Nevertheless, this study has some limitations. Firstly, due to its cross-sectional nature, it does not allow the assessment of the causal relationship between the studied phenomena. Such studies also fail to record changes over time, which are currently dynamic (due to the recession, inflation, COVID-19, and armed conflict in Europe), and they have a significant impact on the level of food security, especially among the socioeconomically vulnerable subpopulation of the elderly. Secondly, due to the lack of representativeness of the study group (only two provinces, people representing senior citizen clubs), the results of the study cannot be applied to the entire population of elderly residents in Poland. This is because there are different cultural and economic contexts among the regions of Poland, and the economic, psychosocial, and health status of socially active seniors (beneficiaries of senior clubs) differs in comparison with socially withdrawn and disabled seniors living in different local communities; thus, this is also a limitation of the study sample. Moreover, in the study group, there was an overrepresentation of women (74.2%) in relation to the Polish population of older people (58.1%) [52], which may result from their greater social activity. The arbitrary selection of study group participants, and the resulting deviations from the characteristics of the general population of older people, may therefore limit the possibility of reproducing the results in a representative group. Despite the limitations of this study, as indicated above, the results noted the great potential of nutritional risk as a construct when studying factors concerning food insecurity. At the same time, individual components of this construct may be useful in the development of strategies aimed at reducing nutritional risk and increasing the food security of the elderly.

## 5. Conclusions

The results of the study confirmed the association between food insecurity and nutritional risk, as well as between the severity of the nutritional risk, the presence of various risk factors, and the characteristics of the study group. Individuals with low food insecurity and high nutritional risk displayed behaviors that may favor malnutrition (i.e., skipping meals, avoiding or limiting food, limited consumption of meat, poultry, fish, eggs, legumes, beverages, fruits and vegetables, dairy products), impaired quality of life (i.e., limited consumption of meals in the company of others, problems with buying food, little appetite), and impaired health (i.e., change in body weight, difficulty in swallowing). This group experienced more nutritional risk factors and concerns about food availability, in addition to being more affected by a lack of basic food products, resulting in reduced meal sizes and skipping meals. These problems occurred more often among people living in an urban environment, alone or with family but without a partner, people suffering from a disease that impedes mobility, people over 75 years of age, and those aged 60–65 years. The obtained results are not always consistent with those from previous studies, but they may confirm the high level of dynamic changes occurring in the living environments of older people. This points to the need for systematic research into nutritional risk factors and feelings of food insecurity in elderly groups in order to diagnose the determinants of their nutritional situation. Although the results pointed to the great potential of nutritional risk as a construct when studying factors of food insecurity, further studies using this approach among representative groups are needed to confirm the results of this study.

The obtained results can find application in the planning of social and health policies and health policies for the elderly. Decision makers in public health policy planning should take nutritional risk and its components into account to increase the effectiveness of actions aimed at reducing food insecurity and negative changes in the health and quality of life of the elderly. In addition, the results of the study can be applied to the further development of conceptual models related to health in this population group.

## Figures and Tables

**Table 1:** Characteristics of the study group.

Characteristics		N = 417	%
Gender	Woman	312	74.8
	Man	105	25.2
Age	60–65 years	94	22.5
	66–70 years	141	33.8
	71–75 years	95	22.8
	Above 75 years	87	20.9
Place of residence	Rural area	122	29.3
	Urban area	295	70.7
Family status	Living alone	154	36.9
	Living only with a partner	168	40.3
	Living with family (with or without a partner)	95	22.8
Financial status	Below average	56	11.3
	Average	308	55.6
	Above average	53	33.1
Education	Vocational and below	149	35.7
	Secondary	143	34.3
	Higher	125	30.0
Subjective health assessment	Worse than peers	84	20.1
	Same as peers	249	59.7
	Better than peers	84	20.2
BMI	Normal	113	27.1
	Overweight	204	48.9
	Obese	100	24.0
Incidence of metabolic disease	No	199	47.7
	Yes	218	52.3
Incidence of a disease that impedes mobility	No	329	78.9
	Yes	88	21.1
Assessment of family relations	Very good	172	41.2
	Good	183	43.9
	Average and bad	62	14.9

**Table 2:** Characteristics of the extracted clusters.

Characteristics	Total	Cluster	
		I Low Food Security and High Nutritional Risk	II High Food Security and Low Nutritional Risk
N	417	180	237
Number of risk factors	6.9; 2.05	8.9 a; 1.15	5.5 b; 1.24
Concerns about food availability *	1.2; 0.50	1.3 a; 0.62	1.1 b; 0.37
Lack of basic food products *	1.2; 0.47	1.2 a; 0.51	1.1 b; 0.44
Reduced meal sizes *	1.2; 0.48	1.3 a; 0.62	1.1 b; 0.30
Skipping meals *	1.3; 0.68	1.5 a; 0.79	1.2 b; 0.53

* answers: 1—no, 2—rarely, 3—at least once a week a, b—mean values denoted by different letters are significantly different *p* < 0.05 (the U Mann–Whitney test).

**Table 3:** Characteristics of the clusters, taking into account selected characteristics of the studied group.

Characteristics		Cluster		*p* (Chi-Square Test)
		I Low Food Security and High Nutritional Risk	II High Food Security and Low Nutritional Risk	
All group		43.2	56.8	
Age	60–65 years	48.9	51.1	0.024
	66–70 years	43.3	56.7	
	71–75 years	30.5	69.5	
	Above 75 years	50.6	49.4	
Place of residence	Rural area	34.4	65.6	0.021
	Urban area	46.8	53.2	
Family status	Living alone	49.4	50.6	0.014
	Living only with a partner	34.5	65.5	
	Living with family (with or without a partner)	48.4	51.6	
Financial status	Below average	59.6	40.4	0.046
	Average	42.2	57.8	
	Above average	39.1	60.9	
Assessment of family relations	Very good	35.5	64.5	<0.001
	Good	43.7	56.3	
	Average and worse	62.9	37.1	
Incidence of a disease that impedes mobility	No	39.5	60.5	0.004
	Yes	56.8	43.2	

**Table 4:** Incidence of nutritional risk factors in clusters.

Factors That Increase Nutritional Risk	Total	Cluster		*p* (Chi-Square Test)
		I Low Food Security and High Nutritional Risk	II High Food Security and Low Nutritional Risk	
Difficulty in chewing or biting	79.9	76.7	82.3	0.157
Loss of appetite	69.5	82.2	59.9	<0.001
Skipping meals	60.4	81.7	44.3	<0.001
Perception of body weight as abnormal	59.2	78.3	44.7	<0.001
Change in body weight	55.9	69.4	45.6	<0.001
Avoiding or limiting food	54.0	67.8	43.5	<0.001
Limited consumption of meals in the company of others	49.9	66.7	36.3	<0.001
Limited consumption of meat, poultry, fish, eggs, and legumes	48.4	66.1	35.0	<0.001
Negative view of food preparation	38.8	52.8	28.3	<0.001
Limited beverage consumption	38.8	52.8	28.3	<0.001
Participation of others in the preparation of meals	33.1	38.3	29.1	0.048
Problems with buying food	29.0	40.6	20.3	<0.001
Use of meal replacements, such as nutridrinks	19.9	31.7	11.0	<0.001
Limited consumption of fruits and vegetables	18.5	23.3	14.8	0.026
Difficulty in swallowing	16.5	25.6	9.7	<0.001
Limited consumption of dairy products	13.7	19.4	9.3	0.003
Change of weight without intention	10.1	12.8	8.0	0.110

**Table 5:** Characteristics of nutritional risk in the study group.

Factors That Increase Nutritional Risk	Total	Increasing Nutritional Risk			Number of Nutritional Risk Factors			
		High Risk (<50 Points)	Low Risk (50 Points and Over)	*p*	Small I Tercile	Average II Tercile	Large III Tercile	*p*
Difficulty chewing or biting	79.9	75.2	95.7	<0.001	82.1	82.8	70.8	0.042
Loss of appetite	69.5	70.3	67.0	0.546	50.9	72.7	84.4	<0.001
Skipping meals	60.4	67.5	36.2	<0.001	36.6	61.2	86.5	<0.001
Perception of body weight as abnormal	59.2	71.2	18.1	<0.001	37.5	57.4	88.5	<0.001
Change in body weight	55.9	62.2	34.0	<0.001	34.8	56.9	78.1	<0.001
Avoiding or limiting food	54.0	59.4	35.1	<0.001	30.4	56.5	76.0	<0.001
Limited consumption of meals in the company of others	49.4	54.2	33.0	<0.001	24.1	52.2	72.9	<0.001
Limited consumption of meat, poultry, fish, eggs, and legumes	48.4	56.3	21.3	<0.001	23.2	50.7	72.9	<0.001
Negative view of food preparation	38.8	44.9	18.1	<0.001	22.3	39.7	56.3	<0.001
Limited beverage consumption	38.8	42.1	27.7	0.011	20.5	38.3	61.5	<0.001
Participation of others in the preparation of meals	33.1	35.6	24.5	0.043	33.0	27.3	45.8	0.006
Problems with buying food	29.0	31.9	19.1	0.017	12.5	31.1	43.8	<0.001
Use of meal replacements, such as nutridrinks	19.9	24.5	4.3	<0.001	0.9	22.5	36.5	<0.001
Limited consumption of fruits and vegetables	18.5	14.9	30.9	<0.001	13.4	17.7	26.0	0.059
Difficulty in swallowing	16.5	19.8	5.3	<0.001	5.4	16.3	30.2	<0.001
Limited consumption of dairy products	13.7	12.4	18.1	0.157	7.1	13.4	21.9	0.009
Change of weight without intention	10.1	11.5	5.3	0.084	5.4	10.5	14.6	0.084

**Table 6:** The severity of nutritional risk using selected characteristics of the study group.

		Total	Nutritional Risk		*p* (Chi-Square Test)	Average Value *
			High (<50 Points)	Low (50 Points and Over)		
All group		100.0	77.5	22.5		44.6; 6.67
Gender	Woman	74.8	74.3	76.6	0.652	44.5 a; 6.87
	Man	25.2	25.7	23.4		44.6 a; 6.03
Age	60–65 years	22.5	22.3	23.4	0.972	45.1 a; 6.00
	66–70 years	33.8	33.7	34.0		45.1 a; 6.11
	71–75 years	22.8	22.6	23.4		44.6 a; 7.23
	Above 75 years	20.9	21.4	19.1		43.4 a; 7.48
Place of residence	Rural area	29.3	26.9	37.2	0.053	45.5 a; 7.17
	Urban area	70.7	73.1	62.8		44.3 a; 6.42
Family status	Living alone	36.9	39.6	27.7	0.059	43.4 a; 6.37
	Living only with a partner	40.3	37.5	50.0		46.2 b; 6.08
	Living with family (with or without a partner)	22.8	22.9	22.3		43.8 a; 7.61
Financial status	Below average	11.3	12.7	6.4	0.043	43.0 a; 7.58
	Average	55.6	57.0	51.1		44.6 a; 6.00
	Above average	33.1	30.3	42.6		45.3 a; 7.32
Education	Vocational and below	35.7	35.3	37.2	0.259	44.6 a; 7.06
	Secondary	34.3	36.2	27.7		45.1 a; 5.71
	Higher	30.0	28.5	35.1		44.1 a; 7.20
Subjective health assessment	Worse than peers	20.1	22.3	12.8	0.119	42.7 a; 7.05
	Same as peers	59.7	58.5	63.8		44.8 b; 6.50
	Better than peers	20.2	19.2	23.4		45.9 b; 6.42
BMI	Norm	27.1	21.7	45.7	<0.001	47.2 a; 7.10
	Overweight	48.9	50.8	42.6		43.9 b; 6.35
	Obese	24.0	27.6	11.7		43.3 b; 6.03
Incidence of metabolic disease	No	47.7	43.7	61.7	0.002	45.6 a; 7.07
	Yes	52.3	56.3	38.3		43.7 b; 6.16
Incidence of a disease that impedes mobility	No	78.9	76.2	88.3	0.011	45.3 a; 6.44
	Yes	21.1	23.8	11.7		42.2 b; 6.98
Assessment of family relations	Very good	41.2	36.8	56.4	<0.001	45.9 a; 6.70
	Good	43.9	45.5	38.3		44.4 a; 6.29
	Average and worse	14.9	17.6	5.3		41.8 b; 6.83

* average nutritional risk score, range 0–64 points. a, b—mean values denoted by different letters are significantly different *p* < 0.05 (the U Mann–Whitney test or the Kruskal–Wallis test).

## Data Availability

Data presented in this study are available on request from the corresponding authors.
